# Eosinophil: An innate immune cell with anti‐filarial vaccine and biomarker potential

**DOI:** 10.1002/hsr2.1320

**Published:** 2023-06-05

**Authors:** Alexander Kwarteng, Caleb Mensah, Priscilla Osei‐Poku

**Affiliations:** ^1^ Department of Biochemistry and Biotechnology Kwame Nkrumah University of Science and Technology Kumasi Ghana; ^2^ Kumasi Centre for Collaborative Research in Tropical Medicine Kwame Nkrumah University of Science and Technology Kumasi Ghana

**Keywords:** CLC, eosinophils, filarial infections, galectins, microfilaria

## Abstract

**Background:**

Filarial infections continue to pose a great challenge in endemic countries. One of the central goals in the fight against human filarial infections is the development of strategies that will lead to the inhibition of microfilariae (mf) transmission. Keeping mf under a certain threshold within endemic populations will stop transmission and eliminate the infection.

**Method:**

A narrative review was carried out to identify the possibilities and limitations of exploring the use of eosinophil responses as an anti‐filarial vaccine, and biomarker for the detection of filarial infections. An extensive literature search was performed in online scientific databases including PubMed Central, PubMed, BioMed Central, with the use of predefined search terms.

**Results:**

A better understanding of the parasite‐host interactions will lead to the development of improved and better treatment or vaccine strategies that could eliminate filariasis as soon as possible. Highlighted in this review is the explorative use of eosinophil‐producing CLC/Galectin‐10 as a potential biomarker for filarial infections. Also discussed are some genes, and pathways involved in eosinophil recruitments that could be explored for anti‐filarial vaccine development.

**Conclusion:**

In this short communication, we discuss how eosinophil‐regulated genes, pathways, and networks could be critical in providing more information on how reliably a front‐line immune player could be exploited for anti‐filarial vaccine development and early infection biomarker.

## 1 INTRODUCTION

Filariasis is an infectious disease caused by threadlike parasitic worms that inhabit the lymphatics, subcutaneous skin, and pleural cavities of infected host.^1^ Within the host, filarial nematodes release millions of microfilariae (*mf*), which circulate periodically in the peripheral blood.^2^ Circulatory microfilariae are the most important stage of the parasite cycle with regard to infection transmission. Therefore, targeting *mf* is crucial if transmission of filarial infection is to be reduced or controlled.^3^ The Circulating filarial antigens (CFAs) test identifies individuals infected with adult worms but does not show microfilaremia, that is, CFA+ and MF−.^4^, ^5^ In endemic regions, about 50% of individuals with latent infections exist, that is, presence of adult worms, yet never develop pathological changes which most often than not are associated with the presence of circulating microfilariae.^6^ In animal models, the successful establishment of immunization strongly suggests differential immune responses in these two disease phenotypes: latency and patency.^7^, ^8^ The molecular mechanisms and pathways involved in the infection stages: patency and latency, are yet to be delineated. Till now, the mechanisms by which filarial nematodes are killed by the mammalian host are not fully known. Nevertheless, trafficking of signals that control the movement of distinct subsets of immune cells into and out of specific tissue is extremely important in immunity, because the accumulation of leukocytes in tissues contributes to a wide variety of diseases.^9^

## 2 LIFE‐CYCLE OF FILARIAL PARASITES

**Figure 1:**
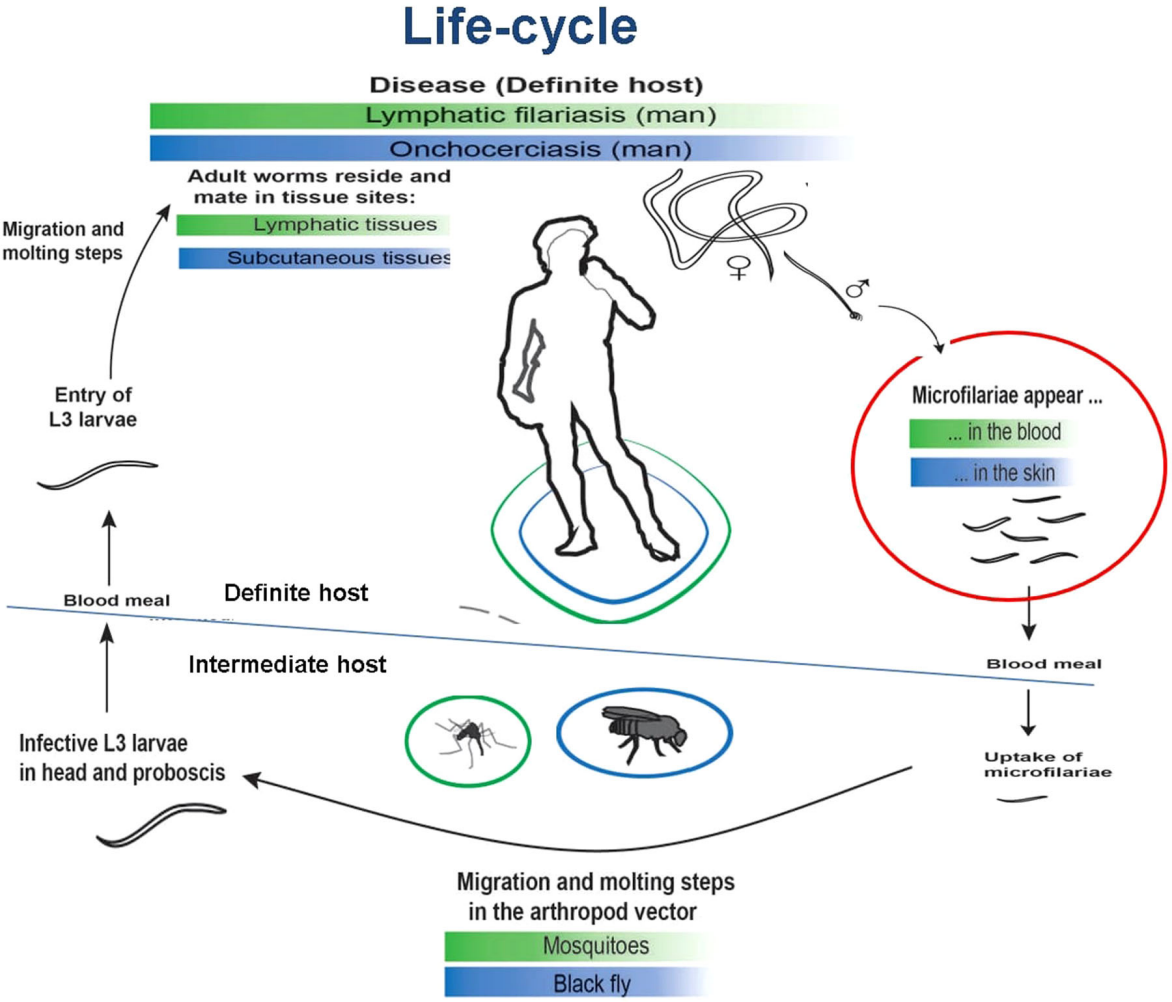
Life‐cycle of filarial parasites. In the event of a blood meal, infective larvae (L3) are transmitted by distinct vectors of filarial worms to human hosts. L3 migrate to specific locations where they develop into adult male and female worms in a time span of 12 months. After mating, fertilized females produce first‐stage larvae (mf), which are then released into the blood or skin, depending on the filarial species. Released mf are subsequently ingested by species‐specific vectors, where they undergo several developmental stages.

The life cycle of filarial worms is convoluted with multiple distinct morphological stages in both vector and mammalian hosts (Figure 1). The cycle begins in the event of an infected female vector taking a blood meal from a human host. Consequently, there is transfer of infective larvae (known as L3) from the vector to the dermis of the host. The vectors for lymphatic filariasis and onchocerciasis are mosquitoes and black flies, respectively. These vectors penetrate the superficial layers of the skin with their proboscis, after which the released larvae commence migration and development into further larval stages. Eventually, they will mature into adult worms in the body over a time span of 6–12 months. Over time, fertilized female worms produce first‐stage larvae termed microfilariae (*mf*), which have an estimated lifespan of 1.5 years. Subsequent biting on the host by the specific vector will lead to the ingestion of *mf* by the vector. Microfilariae enter the stomach of the vector (10–12 days), where most of them get digested and killed. Surviving *mf* molt two times to become infective larvae.^10^ These infective forms migrate to the mouthparts, where they are transmitted to a human host during subsequent blood meals by the vector.

## 3 EOSINOPHILS ARE UTILITY PLAYERS IN FILARIAL INFECTIONS

**Figure 2:**
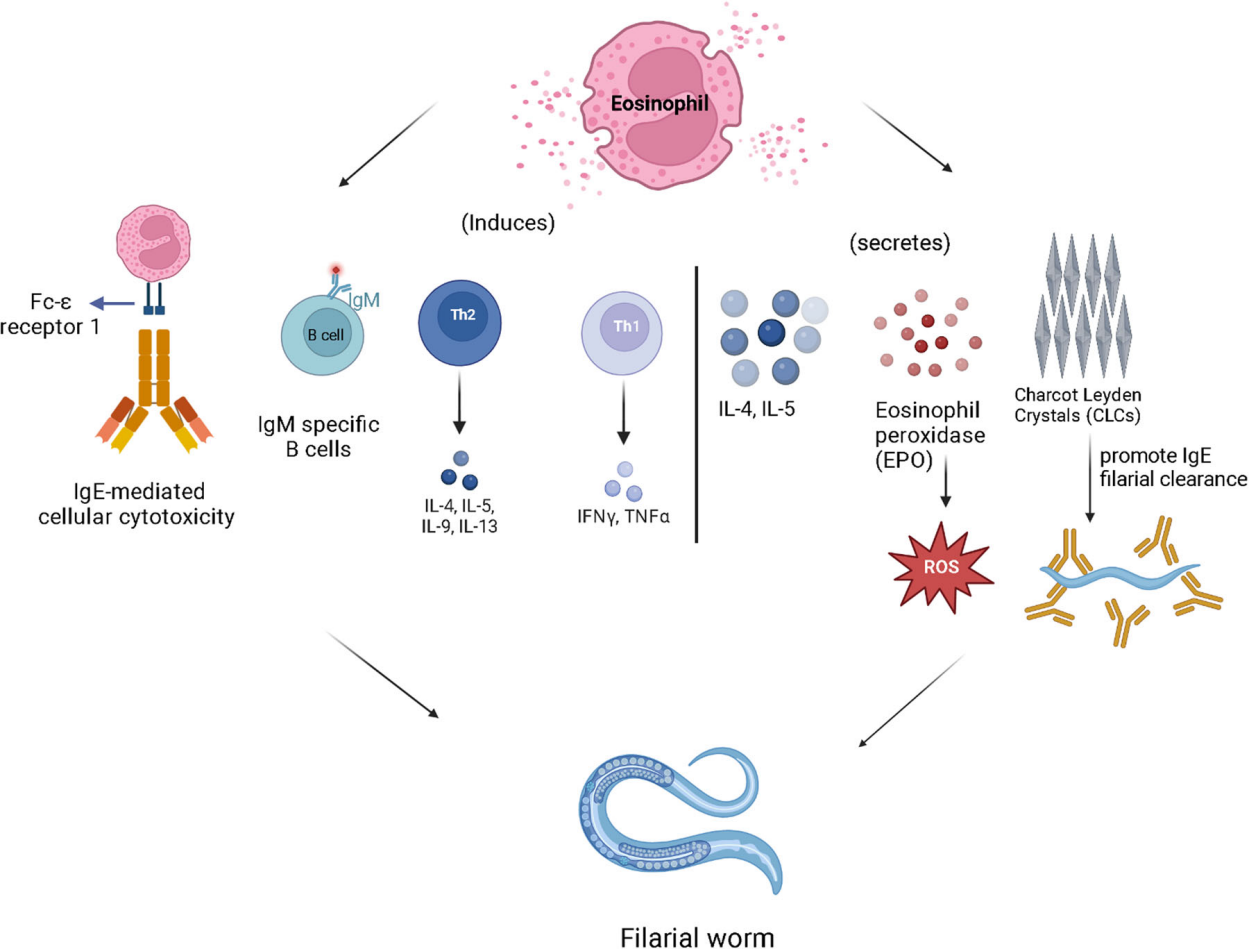
Coordinated protective immune response by eosinophil against filarial infections. Eosinophils secrete IL‐4, IL‐5, and eosinophil peroxidase (EPO), known to promote protective immunity against filarial infections, and Charcot–Leyden crystals, with potential dual roles, that is, protective and immunosuppression. Interaction of the highly expressed Fc‐ε receptor 1 on eosinophil cell surface with the Fc portion of specific‐IgE bound to the filarial parasite promotes cellular cytotoxicity by eosinophils. Activated eosinophil further leads to the induction of IgM‐specific B cells, T helper 1, and 2 cells (Th1, Th2), respectively, which establishes its anti‐filarial protection further. This figure was created with BioRender.com.

During nematode infections, innate immune cells, such as eosinophils, are activated and recruited to the site of infection by a coordinated sequence of events, unlike bacteria infections where early innate responses are predominantly mediated by neutrophils and monocytes. Protective immunity in *Onchocerca volvulus* is eosinophil‐dependent under the regulation of Th2 as well as TLR4.^11^, ^12^, ^13^ Although eosinophils are known to be primary inducers of Th2 responses, their ability to promote Th1 and immunoregulatory cytokines has been established.^14^ Activated eosinophils release IL‐4 and IL‐5 cytokines in addition to other mediators which promote immunity against helminths.^15^ Indeed, the transit of activated eosinophils from the bloodstream to the site of infection has been shown to involve selectins and integrins. Being one of the key players during parasitic infections, eosinophils have also been found to promote humoral responses by priming B cells for the production of antigen‐specific IgM.^16^ Eosinophils promote protective immunity in filarial infections (Figure 2) and as such, contribute significantly to curbing filarial infections.^17^ The granulocytes contain granules and are the cells that attack extracellular large parasites. This hypothesis is supported by studies on other cytotoxic factors, such as eosinophil peroxidase (EPO) and major basic protein‐1 (MBP‐1), whose importance were reported using filarial murine model by Specht et al.^18^ Here, we are also of the view that in peripheral blood, eosinophils could be explored as a better marker for detecting *mf*‐positive patients, since active migration of activated eosinophils through the bloodstream is a characteristic of early filarial infection.

## 4 EOSINOPHILS: SOME LESSONS FROM ANIMAL MODELS AND HUMAN STUDIES

Evidence from both animal models and human studies shows that adult worms significantly elicit differential immune‐suppressive mechanisms, ensuring a moderate milieu for the *mf* at the moment of their release.^2^ Indeed, microfilariae‐positive mice might be considered as those in which immune suppression by the adult worms has been more efficient, whereas their *mf*‐negative counterparts have been able to circumvent suppression and elicit efficient immune responses killing *mf*.^19^ In *O. volvulus* infection, eosinophil infiltration in nodules is dependent on *mf* released from adult worms^20^ and has been shown to target skin‐residing *mf*, which possibly reflects their functional role in host defense strategy.^21^ During inflammation, activated lymphocytes and other immune cells such as neutrophils, dendritic cells, and most importantly natural killer cells migrate to site of infection. In another murine study between adult *Brugia malayi* and microfilariae, it was observed that microfilariae caused reduced leukocytes infiltration into the peritoneal cavity when compared to adult implanted mice.^22^ In human onchocerciasis, it has been shown that infiltration of eosinophils into nodules is dependent on microfilarial release from adult worms, and these cells have been shown to actively attack microfilariae.^20^, ^21^, ^23^

So far, the role of eosinophils seems controversial.^24^, ^25^ But several evidence in mice suggest that protective immunity against *O. volvulus* is Th2 response dependent mediated by eosinophils and IgE.^11^ This further strengthens the point that eosinophil is vital in the destruction of microfilariae, thus promoting filarial immunity. Further studies with irradiated L3 larvae resulted in parasite clearance during subsequent infection; nevertheless, depletion of eosinophils during an immune challenge diminished the protective immunity.^11^ Results from *Onchocerca onchegi* infection in cattle further support the functional capacity of eosinophils in killing parasites.^26^ Elsewhere, it has been documented that in the absence of eosinophils and IL‐5, a transient delay was observed in the development of *Litomosoides sigmodontis*.^27^

## 5 EOSINOPHIL RECRUITMENT: GENE REGULATION

**Figure 3:**
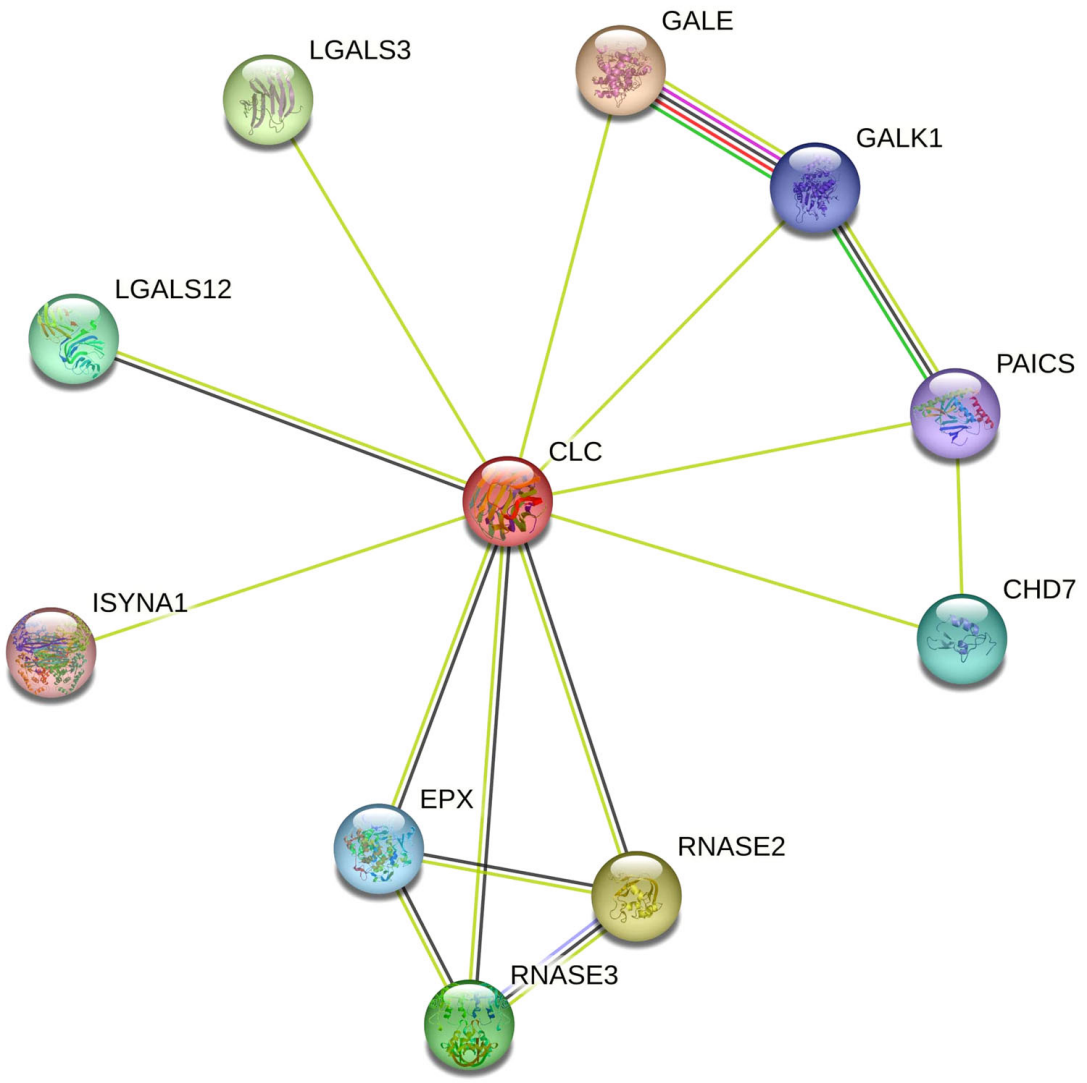
Functional association network of eosinophil‐associated genes involved in eosinophil‐mediated immunity, and eosinophil migration to site of infection, with CLC as a reference point. Color‐coded lines represent specific associations. Purple lines represent experimentally determined interactions. Green connectors indicate gene neighborhood, red lines indicate gene fusion, and black indicates co‐expression. Sheen green lines represent statistically relevant co‐occurring gene names from scientific texts, while the light blue line indicates protein homology (as seen in RNASE2 and RNASE3). The network was generated using STRING database (https://string-db.org/).

In the early stage of filarial infection, there could be increased eosinophil‐associated gene regulation. This is critical as it will lead to the massive recruitment of eosinophils to the site of the nematode infection as well as heightening the signal for other related immune cells to be trafficked to the site of infection. Genes such as *CLC*, *RNASE2*, *ADD3*, *ITGB1*, *ZFAND5*, and *ARGLU1*, among others, are known to be produced by eosinophils in human filarial infections.^28^ Most of the regulated genes support the relevance of innate immune cells during filarial infections. Although the current role of CLC, also known as gelactin‐10, as an eosinophil lysophospholipase is being debated, it is known to possess IgE binding activities, which may contribute to parasite clearance upon IgE interaction^29^, ^30^; however, it must be indicated that in other nonparasitic‐related diseases, CLC may also be produced and is discussed in detail in subsequent sections. CLC is profoundly produced and released in activated eosinophils during parasite infections. The regulation of these genes in filarial infection induces an inflammatory response, which leads to the recruitment of early innate immune cells such as eosinophils to site of infection through a coordinated sequence of events that begins with the elaboration of various mediators able to specifically promote their migration from the intravascular compartment. The majority of these regulated genes (Figure 3) promote inflammation, cell migration, cell movement, and most notably eosinophil development and function. Indeed, genes, such as *CTSZ* and *CAT*, are known to heighten innate immune response and offer protection.^31^, ^32^ Genes such as *CLC* and *RNASE2* are crucial in eosinophils development and functionality. Interestingly, *CLC* has been shown to possess high binding activity to IgE,^29^ an important immunoglobulin in parasite biology with a definitive role in parasite clearance, which is carefully regulated by the immunoregulatory IgG4.^33^ Elsewhere, eosinophils‐generated superoxide radicals have been shown to destroy helminth cuticles.^34^, ^35^

## 6 EOSINOPHIL RECRUITMENT: PATHWAYS INVOLVED

Recruitment of eosinophils is complicated and could involve pathways such as the Cdc42 signaling, Actin nucleation by ARP‐WASP complex, Rac signaling, CD28 signaling in T Helper cells, Actin cytoskeleton signaling, Superoxide radicals, Signaling of Rho Family GTPases, and Ephrin receptor.^28^ It can be inferred that elevated regulation in integrin, eosinophil associated genes could suggest significant innate immune development and differentiation as well as coordinated immune cell trafficking as host response to filarial infections.

RNAseq profiling of the various filarial infection phenotypes could offer more comprehensive insight into involved regulated genes, pathways, networks, and possible signaling events in the innate immune response to filarial nematodes. Filarial infections show increased expression of these eosinophil‐induced cell migration and cell movement. Regulating inflammatory responses is extremely essential so far as immunity is concerned. Although filarial parasites are generally immunosuppressing, others have identified these parasites to induce potent inflammatory responses.^22^

## 7 EOSINOPHIL RECRUITMENT: COULD INTEGRINS BE SUITABLE TARGET?

Integrins are important in eosinophils migration. A review by Abram and Lowell^36^ associated increased oxidative burst with integrin activation. One of the prominent pathways found to be highly regulated during filarial infection is Cdc42. Recently, the Cdc42 pathway has been shown to promote host defense against fatal infection.^37^ Loss of Cdc42 leads to massive impaired directed migration in fibroblasts and astrocytes^38^ and also directly or indirectly regulates RhoA.^39^ Further, Cdc42 has been shown to contribute significantly to integrin‐mediated activation of the lipid kinase PI 3‐kinase, the protein kinases Akt and Cdc42‐associated tyrosine kinase‐2 (ACK‐2) and MAPK.^40^

Regulation of the Rho GTPases induces the assembly of contractile actin and myosin filaments,^41^, ^42^ which is crucial during cell migration. In filarial‐infected subjects, it is possible that the local host‐parasite interactions that induce and condition adjacent endothelial cells favor the recruitment of functional and relevant eosinophils primarily to site of infection. Accumulating evidence shows that eosinophils also express Rho‐related GTPases such as Rac1 and Rac2, which play an essential role in the release of superoxide as well as regulate actin remodeling.^43^, ^44^, ^45^ Rac1 has been implicated in MAPK and JNK activation and Rho in the activation of MAPK, FAK, and phosphatidylinositol 4‐phosphate (PtdIns4P) 5‐kinase.^46^ Interestingly, filarial parasites possibly activate the eosinophil‐associated pathway, which in turn activates several GTPases, resulting in the mobilization of potent granules which are toxic to filarial parasites.

The targeting of integrins and their binding partners with anti‐inflammatory drugs and inhibitors has attracted much attention in the clinic. In several clinical trials, the use of monoclonal antibodies, such as Natalizumab, against some integrins, has been successful in the treatment of multiple sclerosis^47^ and Crohn's disease,^48^ whereas blocking all α4‐integrin‐mediated leukocyte trafficking leads to an increased susceptibility to infection. Indeed, this clinical achievement has established the “proof of principle” that specifically interfering with leukocyte migration into tissue is an effective and new therapeutic strategy, and could also be explored in filarial biology, especially among individuals presenting with chronic filarial infections.

## 8 EOSINOPHILS AND EOTAXIN‐1–CCR3 AXIS

Recruitment of eosinophils involves a signaling cascade in which secreted chemokines interact with heterotrimeric G protein‐coupled receptors (GPCRs) and especially with C‐C chemokine receptor type 3 (CCR3), the receptor for eotaxins.^49^ In their study, El‐Shazly et al. documented that the binding of eotaxins to CCR3 induces a signaling cascade, which is accompanied by calcium mobilization and the activation of Ras‐MAPK, especially the extracellular signal‐regulated kinase 1 and 2 (ERK‐1/2) dependent pathways.^49^ CCR3‐deficient mice have a profound defect in eosinophil migration,^50^ and blocking of CCR3 impairs eosinophil trafficking.^51^

In a mouse model, CCR3 expression on eosinophils promoted L3 larvae killing in *O. volvulus* infection,^11^ whereas CCR3 knockout mice sustained elevated muscle larvae burden in *Tricuris spiralis* infection.^52^ However, the expression of CCR3 is not restricted to eosinophils alone, but also in cells such as neutrophils^53^ and basophils.^54^ The activation of this signaling pathway indicates migration of eosinophils to site of infection. In a study by Gentil et al. where a mouse model of filarial infection was used, the authors found that eotaxin‐1 (chemokine, which binds to CCR3) was found to modulate the activation of inflammatory cells leading to helminth clearance,^55^ thus further confirming the protective functions of eosinophils during filarial infection. However, eosinophil activity in another study demonstrated their contribution to *L. sigmodontis* development,^27^ a scenario that needs to be carefully considered in human vaccine programs to control helminth infections. Together, these studies show direct evidence that the activation and recruitment of eosinophils and their secreted proteins could be linked with protection since eosinophils in lymphatic filariasis and onchocerciasis were significantly expressed in subjects without *mf*; hence suggesting their importance in determining filarial infection outcome.

## 9 EOSINOPHILS RELY ON PREFORMED POISONS

Granulocytes are generated from hematopoietic stem cells and subsequently differentiate into myeloid progenitor lineages. In fact, in circulating leukocytes of healthy humans, granulocytes consist of approximately 50% neutrophils, whereas eosinophils and basophils make up 2%–5% and 1%, respectively. Largely, these cells are considered either friends or foes of helminths because they are normally induced during helminth infections.^56^ The role of granulocytes in filariasis appears to be diverse. They are believed to either promote protective immunity or even facilitate parasite establishment. Interestingly, eosinophils are not only associated with helminth infections but are hallmarks of allergic responses, asthma, and viral infections too. Peripheral eosinophil counts may reach up to 75% during filarial infections and can induce tropical pulmonary eosinophil in *W. bancrofti‐* and *B. malayi*‐infected individuals. Eosinophils contribute to the destruction of helminths by antibody‐dependent cellular cytotoxicity (ADCC),^57^ an innate immune mechanism.

One of the tenets of eosinophils is that they contain preformed granules which makes them extremely competent as innate immune leucocytes. By this feature, it offers the cells the ability to respond on time at the onset of infection. It is well documented that activated eosinophils release granule proteins, such as Ribonuclease (RNAS2 and RNASE3), eosinophil cationic protein (ECP), MBP, and EPO following nematode infections. Studies in EPO and MBP knockout mice have demonstrated that, through their granule contents, eosinophils facilitate *L. sigmodontis* larval clearance since in their absence worms develop faster.^18^ Others have suggested that eosinophils are essential for early worm development.^27^

## 10 EOSINOPHIL AND CHARCOT–LEYDEN CRYSTAL: PRIMARY BIOMARKER FOR FILARIAL INFECTIONS?

In addition to the above granules, activated eosinophils release carbohydrate‐binding proteins called galectins. Galectins are β‐galactoside‐binding animal lectins and are characterized by conserved amino acid sequences in the carbohydrate recognition domain with high affinity for β‐galactosides.^58^ Currently, 15 galectins have been characterized and although primarily localized in the cytoplasm or extracellular space, under certain physiological conditions, they can translocate into the nucleus or associate with intracellular vesicles.^58^ While galectins may not have specific individual receptors, each can bind to a set of cell‐surface glycoproteins containing suitable oligosaccharides through lectin‐carbohydrate interactions.^59^

Eosinophils are a potential source of Charcot–Leyden crystal (CLC)/Gal‐10, as it constitutes approximately 10% of the total cellular protein in human eosinophils.^60^ CLC/Gal‐10 appears to have a dual role with both protective and immunosuppressive effects. CLC/Gal‐10 has been reported to possess IgE binding activities^29^ and although there is currently no available data, this may contribute to filarial clearance upon IgE binding.

On the other hand, in another cell type, CLC/Gal‐10 protein appears to also have immunosuppressive functions, since it enhanced the suppressive capacity of regulatory T cells.^61^ Elsewhere, increased IL‐10 and TGF‐β are believed to be highly produced in patently infected individuals.^62^ Posttranslational modifications may influence the conversion of *CLC/Gal‐10* mRNA into proteins (splice variants) in the presence of *W. bancrofti* and *O. volvulus* infection as demonstrated by a proteomics study where three variants of CLC/Gal‐10 protein were identified.^61^ The existence and functions of possible isoforms of CLC/Gal‐10 proteins during filarial infections are not known and further investigation of this molecule may be warranted.

Furthermore, galectin‐9 from *Toxacaris leonina* shares 35% homology with human galectin‐9 and recombinant forms lead to diminished intestinal inflammation in mice via increased IL‐10 and TGF‐β.^63^ In fact, the development and functional capacity of eosinophils are enhanced by the Th2‐producing cytokine IL‐5, chemotaxis‐related CCR3,^15^ as well as the CLC, a lysophospholipase that possesses IgE binding activities.^29^ Parasite‐specific IgE is elevated in latently infected compared to their patent counterparts, where the immunoregulatory IgG4 is predominantly expressed.^33^, ^64^, ^65^ Collectively, these previous reports give rise to evidence that the galectin family of proteins may have intrinsic functions associated with the immunomodulation of host responses and this should be exploited in filarial infections to fully characterize the underlying mechanisms. The role of CLC/Gal‐10 protein continues to be debated and CLC as a potential diagnostic marker awaits to be investigated. While it is believed to have protective immunity, a study by Kubach et al. has attributed it to regulatory functions.^61^ In filariasis, individuals without *mf* have enhanced adaptive immune responses, while the presence of *mf* is associated with immunosuppression. Thus, the exact role of CLC/Gal‐10 proteins is yet to be comprehensively documented. However, it appears to be highly regulated in both lymphatic filariasis and onchocerciasis and thus could serve as a reliable biomarker at the messenger and protein levels.

Since the deposition of CLCs is linked to eosinophil activation and infiltration, they can be associated with not just filarial infections, but a variety of eosinophilic diseases including myeloid leukemia, asthma, and allergic diseases.^66^, ^67^ Some studies have identified CLC/Gal‐10 as a potential biomarker of eosinophil engagement in allergic rhinitis, asthma, and other diseases associated with eosinophil activation.^66^ A study by Chua et al. identified CLC/Gal‐10 as a potential surrogate biomarker of eosinophilic airway inflammation in induced sputum, which could aid in asthma treatment and management.^68^ Elsewhere, elevated CLC/Gal‐10 levels have been measured as a biomarker of active eosinophilic inflammation that strongly correlates with the number of esophageal eosinophils in eosinophilic oesophagitis.^69^ A different study on celiac disease found CLC/Gal‐10 expression to be related to both disease activity and tissue eosinophils population in intestinal lesions, suggesting CLC/Gal‐10 as a potential biomarker for assessing tissue damage and eosinophil participation in the pathogenesis of gluten intolerance.^70^ These studies indicate the possibility of exploring site‐specific CLC/Gal‐10 deposition during filarial manifestations as a possible biomarker for assessing the progression of filarial infection.

## 11 EOSINOPHILS: WHAT THE OMICS HAS TO OFFER!

Current advances in molecular biotechnology put us in a better position to investigate in detail eosinophil‐induced regulated genes, pathways, as well as networks implicated during filarial infections since immune cell trafficking, is extremely vital for ensuring protective immunity during these nematode infections. RNA and single nuclei sequencing seem to be one of the most appropriate techniques today, which could provide deeper and more reliable insight into the underlying eosinophil‐driven mechanisms during filarial infections. Some data appear to be trickling but much is needed to arrive at definitive conclusions on the eosinophils' protective immunity and/or otherwise during filarial infections. More studies on eosinophil‐specific RNA sequencing and single nuclei RNA sequencing will be needed to offer key insight into eosinophils’ role in immunity during filarial infection.

## 12 EOSINOPHILS AND VACCINATION IN FILARIAL INFECTIONS

Amongst the different approaches being discussed is the development of an effective vaccine, although the discussion on the vaccine doses used in the various experiments is out of the scope of this review. Anti‐filarial vaccines would significantly reduce a major health burden in the tropics and could also become a promising tool for the elimination of filarial infections. Indeed effective vaccines have been hypothesized to improve economic development in endemic regions and thus, have a positive impact on health.^71^ In various animal models and livestock, vaccines against nematodes have been successfully tested.^72^ Indeed, the use of two excretory‐secretory antigens from *Heamonchus contortus* in sheep resulted in a significant decrease in fecal egg counts compared to an unvaccinated control group.^73^ The human hookworm vaccine initiative carried out a Phase 1 trial.^74^ In the pursuit of developing a filarial vaccine, studying naturally occurring protective host immune responses against these nematodes may be highly informative.

Eosinophils have been shown to contribute effectively towards the elimination of microfilariae during parasite infection as reviewed^75^, ^76^, ^77^ as well as in vaccination studies.^78^ A recent study by Ehrens et al. elucidated a conserved mechanism by which eosinophils mediate protection against filarial worms. They reported that eosinophils, upon infection by microfilariae and infective L3 larvae of *L. sigmodontis*, release extracellular DNA traps (EETosis) that inhibit microfilariae motility, and promote microfilariae clearance.^79^ It is documented that eosinophils express high‐affinity FC‐epsilon receptor1 for IgE binding.^80^ Interestingly, in a murine model of filarial infection, basophil‐depletion during primary infection of *L. sigmodontis* resulted in lowered eosinophils, serum IgE as well as parasite‐specific T cell responses but did not alter adult worm numbers.^81^ However, in the same study, the authors found no protection of basophils in secondary infection of *L. sigmodontis*.^81^ Several studies have shown that IgE contributes tremendously to parasite killing by acting as a ligand to ADCC by eosinophils and other immune cells.^57^ Thus, it is reasonable to speculate that IgE− mediates eosinophil activation altogether to promote resistance to helminth infections. Several irradiated infective larvae vaccine studies have supported the idea of eosinophil activation being a primary host determinant for filarial clearance.^82^, ^83^ In IL‐5 transgenic mice, overexpression of IL‐5, which leads to a high population of functional eosinophils (eosinophilia), enhanced filarial parasite killing.^84^ Several reports have hinted at the role of eosinophils on humoral, and cell‐mediated immunity. A study by Wong et al. identified the role of eosinophils in the regulation of humoral immunity through their influence on B cell homeostasis and proliferation upon activation.^85^ Similarly, eosinophils are capable of regulating T cell function with regard to polarization of T cells to either the Th1 or Th2 pathway due to their expression of both Th1‐ and Th2‐associated cytokines.^54^, ^86^, ^87^ This suggests that a filarial vaccine engineered to also activate functioning eosinophils will further aid in triggering the adaptive arms of the immune system while performing its function as an anti‐filarial agent.

One possible way of constituting a vaccine construct with an agent that could activate eosinophils is with the inclusion of a constituent that triggers IL‐5 production upon administration. IL‐5 in humans is the primary growth factor for eosinophil maturation, activation, and survival.^88^ With the association of increased IL‐5 production to most human diseases associated with hypereosinophilia,^89^ one may infer it is counterintuitive to consider this cytokine as a possible therapeutic intervention. However, evidence points out that the overexpression of IL‐5 alone appears to be insufficient for inducing eosinophil‐mediated organ dysfunction and damage.^90^ Further studies may be warranted for the use of eosinophil‐inducing agents in vaccine designs against filarial infections to assess the feasibility of this potential.

Despite the conferred protection eosinophils provide during filarial infections, caution must be taken when considering it for vaccine engineering. Eosinophils and their granule proteins have been implicated as mediators of hypersensitivity diseases. For example, there have been established associations between increased eosinophilia and the occurrence of asthma disease and its severity.^91^, ^92^ An abundant eosinophil granule protein, MBP‐1 is a basic protein highly toxic to, and can damage mammalian cells in vitro, but is also known to destroy helminths and bacteria.^93^ Eosinophil‐derived neurotoxin (EDN), another eosinophil granule protein, is documented to have toxic effects on mammalian cells, including myelinated neurons, leading to cellular damage and death.^94^ This effect could implicate EDN presence in the occurrence of neurodegenerative diseases. For example, high levels of serum EDN have been associated with the pathophysiology of amyotrophic lateral sclerosis, a progressive neurodegenerative disease characterized by the depletion of motor neurons in the brainstem, spinal cord, and motor cortex.^95^ Furthermore, eosinophil granule proteins, including MBP, ECP, and EPO, are known to play crucial roles in the pathophysiology of eosinophilic myocarditis.^96^ These concerns must be explored extensively and addressed before a filarial vaccine that is engineered toward eosinophil activation can be considered, especially in filarial‐exposed populations.

## 13 CONCLUSION

A better understanding of host‐parasite interaction will lead to the development of improved and better treatment strategies if the goal to eliminate filariasis is to be achieved soon. Eosinophil is vital in the destruction of microfilariae thus promoting filarial immunity. However, other laboratories have reported some regulatory, and toxic roles of eosinophils on filarial parasite and mammalian host cells, respectively. In this review, we have discussed the prospect of some genes, pathways, and insight into previous vaccine trials. In addition, we also discussed the potential use of eosinophil‐producing CLC/Galectin‐10 as a reliable biomarker both at the mRNA and serum levels. Therefore, there is a lot to learn about this important innate immune cell. To do this, we need to continue to explore the use of high throughput techniques such as RNA sequencing and single nuclei RNA sequencing to throw more light on the role of eosinophils among individuals with filarial infection.

## AUTHOR CONTRIBUTIONS

**Alexander Kwarteng**: Conceptualization; visualization; writing—original draft; writing—review and editing. **Caleb Mensah**: Visualization; writing—review and editing. **Priscilla Osei‐Poku**: Visualization; writing—review and editing.

## CONFLICT OF INTEREST STATEMENT

The authors declare no conflict of interest.

## TRANSPARENCY STATEMENT

The lead author Alexander Kwarteng affirms that this manuscript is an honest, accurate, and transparent account of the study being reported; that no important aspects of the study have been omitted; and that any discrepancies from the study as planned (and, if relevant, registered) have been explained.

## Data Availability

Data sharing is not applicable to this article as no new data were created or analyzed in this study.
